# Impact of the COVID-19 Pandemic on Patients’ Refusal of Emergency Medical Services (EMS) Transport

**DOI:** 10.7759/cureus.104697

**Published:** 2026-03-05

**Authors:** Vijay Reddy, Patrick Loeffler, Peter Bui, William Souter, Bradley M Golden, John McManus

**Affiliations:** 1 Emergency Medicine, Augusta University Medical College of Georgia, Augusta, USA; 2 Family Medicine, Augusta University Medical College of Georgia, Augusta, USA

**Keywords:** ambulance transport, covid-19, covid-19 pandemic, emergency medical services (ems), healthcare utilization, patient refusal, prehospital care

## Abstract

Background

Emergency Medical Services (EMS) activations declined during the coronavirus disease 2019 (COVID-19) pandemic. The reasons patients accept or decline EMS evaluation and transport are multifactorial. This study examined changes in EMS transport refusal rates during the early COVID-19 pandemic compared with the prior year.

Methodology

Run reports were obtained from a single ground ambulance service (SouthStar EMS, North Augusta, South Carolina, USA). Calls during the first 90 days following the U.S. national emergency declaration for COVID-19 (March 13-June 11, 2020) were compared with the same period in 2019. A total of 1,889 calls occurred in 2019, and 1,707 calls occurred in 2020. Runs were categorized as emergent (911) or non-emergent scheduled transports. Outcomes were analyzed using Fisher’s exact test.

Results

Emergent calls increased from 1,148/1,889 (60.8%) in 2019 to 1,301/1,707 (76.2%) in 2020 (p < 0.00001). Refusal of EMS-recommended transport after evaluation increased from 66/1,148 (5.7%) in 2019 to 113/1,301 (8.7%) in 2020 (p = 0.0021). The combined proportion of patients refusing evaluation or refusing recommended transport increased from 189/1,148 (16.5%) to 249/1,301 (19.1%) (p = 0.0376).

Conclusions

During the early COVID-19 pandemic, refusal of EMS-recommended transport increased significantly in this EMS system. These findings suggest that pandemic-related concerns may have influenced patient decision-making regarding prehospital care. Further multi-system studies are needed to better characterize contributing factors and clinical outcomes.

## Introduction

The coronavirus disease 2019 (COVID-19) pandemic substantially altered healthcare utilization across the United States. Multiple studies have demonstrated reductions in emergency department visits and Emergency Medical Services (EMS) activations during the early stages of the pandemic despite ongoing community medical need [1,2]. Fear of exposure and uncertainty regarding hospital safety likely contributed to changes in patient behavior [3].

Refusal of EMS transport is influenced by medical, social, and system-level factors. While some refusals may be appropriate, refusal of recommended transport carries risk, particularly when serious illness or injury is present [4]. Understanding how the pandemic affected refusal patterns is important for EMS operations and public health messaging.

This study evaluated EMS refusal rates during the first 90 days of the declared COVID-19 emergency compared with the same period in the preceding year. We hypothesized that refusal of EMS evaluation and transport would increase during the pandemic period.

## Materials and methods

Study design and setting

This retrospective observational study analyzed EMS run report data from SouthStar EMS, a ground ambulance service operating in North Augusta, South Carolina. Patient encounters were documented using the ESO electronic health record system.

Study period

Two 90-day periods were compared: March 13-June 11, 2019, and March 13-June 11, 2020, beginning with the U.S. national emergency declaration for COVID-19.

Call classification and disposition categories

Runs were classified as emergent (911) activations or non-emergent scheduled transports. Encounter dispositions included transport to a medical facility, cancellation, refusal of evaluation, and refusal of EMS-recommended transport after evaluation on scene.

Study outcomes

The primary outcome was the proportion of emergent (911) encounters resulting in refusal of EMS-recommended transport after evaluation. Secondary outcomes included refusal of evaluation, overall refusal of evaluation or refusal of recommended transport, cancellation rate, and the proportion of emergent versus non-emergent activations during the study periods.

Statistical analysis

Categorical comparisons between 2019 and 2020 were performed using Fisher’s exact test. Statistical significance was defined as a p-value <0.05. Analyses were conducted using SPSS Statistics for Windows, Version 27.0 (IBM Corp., Armonk, NY, USA).

Ethical approval

The Augusta University Institutional Review Board reviewed this study and determined it to be exempt (approval number: 1858049). Informed consent was not required.

## Results

A total of 1,889 EMS calls were recorded during the 2019 study period compared with 1,707 EMS calls during the 2020 pandemic study period. The proportion of emergent (911) activations increased significantly from 1,148/1,889 (60.8%) in 2019 to 1,301/1,707 (76.2%) in 2020 (p < 0.00001).

Among emergent encounters, refusal of EMS-recommended transport after evaluation increased from 66/1,148 (5.7%) in 2019 to 113/1,301 (8.7%) in 2020 (p = 0.0021). Additionally, the combined proportion of patients refusing evaluation or refusing recommended transport increased from 189/1,148 (16.5%) to 249/1,301 (19.1%) (p = 0.0376).

Table 1 presents the total call volumes and disposition outcomes for each study period. Table 2 summarizes the results of Fisher’s exact tests comparing key EMS refusal outcomes between 2019 and 2020.

**Table 1:** EMS call outcomes before versus during the COVID-19 pandemic. EMS = Emergency Medical Services; COVID-19 = coronavirus disease 2019

Outcome category	2019 (n)	2020 (n)
Total calls	1,889	1,707
Emergent (911) calls	1,148	1,301
Cancelled calls	47	86
Refused evaluation	123	136
Refused transport after evaluation (AMA)	66	113
Transported to the hospital	837	866

**Table 2:** Statistical comparisons between 2019 and 2020 EMS outcomes. EMS = Emergency Medical Services

Outcome comparison	2019, n/N (%)	2020, n/N (%)	Statistical test	P-value
Emergent calls out of total EMS calls	1,148/1,889 (60.8%)	1,301/1,707 (76.2%)	Fisher’s exact	<0.00001
Refusal of transport after evaluation (primary outcome)	66/1,148 (5.7%)	113/1,301 (8.7%)	Fisher’s exact	0.0021
Refusal of evaluation or transport (combined outcome)	189/1,148 (16.5%)	249/1,301 (19.1%)	Fisher’s exact	0.0376

## Discussion

This retrospective study identified a statistically significant increase in EMS transport refusal during the early phase of the COVID-19 pandemic. Refusal of EMS-recommended transport after evaluation increased from 66/1,148 (5.7%) in 2019 to 113/1,301 (8.7%) in 2020. These findings suggest that patients were more likely to decline hospital transport despite provider recommendations during the pandemic period.

Our results are consistent with broader reports demonstrating major shifts in emergency care utilization during early COVID-19. Lerner et al. reported nationwide decreases in EMS activations during the initial pandemic months, along with increased deaths on scene, raising concern that patients may have delayed or avoided emergency care [1]. Similar trends were observed internationally, with Siman-Tov et al. describing changes in EMS treatment and refusal patterns during COVID-19 surges [2].

Avoidance of healthcare settings during the pandemic has been widely documented. Niforatos et al. identified barriers to emergency department usage, including fear of infection and uncertainty regarding hospital safety [3]. Such factors may have contributed to the increased refusal rates observed in the prehospital environment.

Transport refusal remains an important operational and clinical challenge for EMS systems. Prior work by Knight et al. demonstrated that refusals of care occur regularly in out-of-hospital practice and may carry risk when patients decline recommended evaluation or transport [4]. Although morbidity and mortality following refusal are generally low in many studies, refusal may delay definitive care for high-risk presentations [5].

Shekhar et al. reported reductions in EMS calls for acute cardiovascular emergencies during early COVID-19, raising concern for delayed care-seeking behavior and downstream adverse outcomes [6]. Pandemic-specific refusal behavior has also been described in other EMS contexts. Glenn et al. reported increased refusals following naloxone administration during COVID-19, suggesting that concerns regarding hospital exposure may have influenced decision-making even in potentially life-threatening conditions [7].

Limitations

This study was conducted within a single EMS system and may not be generalizable to other regions. The retrospective design limits causal inference, and patient-level clinical outcomes, demographics, and motivations for refusal were not available. Regional lockdown policies and public messaging may also have influenced refusal behavior.

Future directions

Future multi-center studies incorporating patient characteristics, chief complaints, and follow-up clinical outcomes would help clarify the drivers and consequences of EMS transport refusal during public health emergencies.

## Conclusions

Refusal of EMS-recommended transport increased significantly during the early COVID-19 pandemic in this EMS system. These findings suggest that pandemic-related concerns may have influenced patient decision-making at the prehospital level. Improved understanding of refusal behavior may support targeted EMS communication strategies and system planning during future public health crises.
